# Introduction to advances in multicomponent plasmonic hybrid nanoarchitectures for versatile applications

**DOI:** 10.1039/d4na90010c

**Published:** 2024-01-29

**Authors:** Hao Jing

**Affiliations:** a Department of Chemistry and Biochemistry, George Mason University Fairfax Virginia 22030 USA hjing2@gmu.edu

## Abstract

An introduction to the *Nanoscale Advances* themed collection on multicomponent plasmonic hybrid nanoarchitectures with precisely tailored properties for emerging applications, featuring burgeoning research on a variety of multifunctional plasmonic nanoparticles with synergistically reinforced properties.

## Abstract 2

Dr Hao Jing introduces the *Nanoscale Advances* themed collection on multicomponent plasmonic hybrid nanoarchitectures for versatile applications.

## Graphical abstract

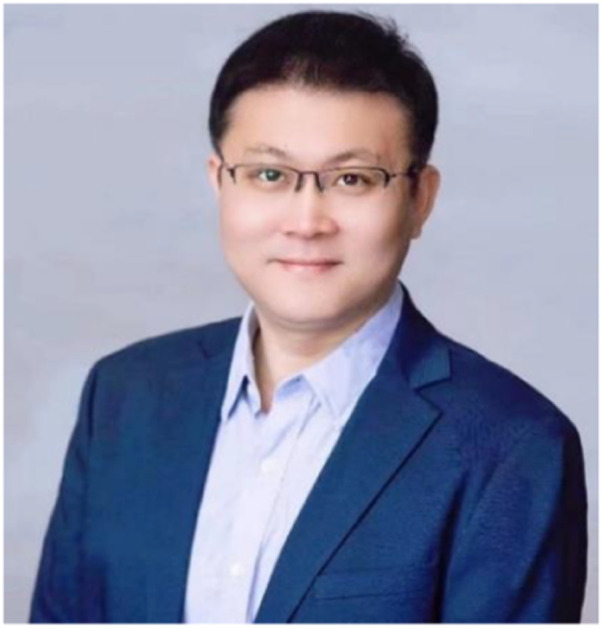

Multicomponent plasmonic hybrid nanoarchitectures composed of two or more condensed nanoscale domains with distinctive material compositions, geometries, and physical dimensions exhibit diverse functions owing to the synergistically enhanced physicochemical properties. Over the past few years, the unique and newly emerging features of multicomponent plasmonic nanostructures, such as finely tuned plasmon resonances, plasmon-induced energy transfer, and enhanced light–matter interactions have been actively investigated by researchers across different disciplines.^1^ Besides, the multicomponent plasmonic structures with unconventional phases and engineered strains/surfaces show great prospects in electrochemical energy storage and conversion, such as electrocatalytic hydrogen and oxygen evolution reaction (HER/OER) due to the abundant structural effects and unique crystal structures.^2^ To maximize the full potential of multicomponent plasmonic hybrid nanomaterials, a myriad of state-of-the-art synthetic approaches, such as seed-mediated heterogeneous nucleation, atomic replacements, ion exchange, absorption on supports, and peptide/DNA-mediated self-assembly, have been exploited to overcome the lattice mismatch between the constituent components for the direct synthesis of colloidal complex heterostructure nanoparticles. Comprehensive studies on the architectural complexity, unprecedented controllability, as well as promising applications in plasmonic photoswitches, photodynamic/photothermal therapy, photoacoustic imaging and colorimetric/chiral sensing, will guide us toward new directions using multicomponent plasmonic hybrid nanomaterials with optimized performances.

Most recently, the enticing potential of structurally well-defined dual plasmonic hybrid hetero nanostructures integrating noble metal and nonstoichiometric copper chalcogenide has attracted significant attention, which results in the expansion of the libraries of multicomponent plasmonic hybrid nanomaterials.^3^ Dual plasmonic nanoparticles with tunable copper vacancies and efficient coupling/crosstalk between two mechanistically distinct localized surface plasmon resonances (LSPRs) have gradually emerged as an intriguing platform of superstructures due to their synergistically reinforced optical and photocatalytic properties.^4^ With deeper understanding of the structure–property relationships, we will open avenues for exploring new types of multicomponent plasmonic nano-entities.

This collection of reviews, minireviews and research articles cover the rational design, synthesis and characterization of multicomponent plasmonic hybrid nanoarchitectures with tailored chemical and physical properties, as well as their utilization in a wide variety of applications. The rational design and the structural optimization of plasmonic antenna-reactor hybrid nanomaterials with dual functions, can shed light on the interplay between plasmonic and catalytic effects experimentally. Wang *et al.* (https://doi.org/10.1039/D3NA00498H) develop a multistep synthetic approach to assemble colloidal Au@C/Pt core@shell/satellite supra-nanostructures, in which the Au core functions as a light-harvesting plasmonic nanoantenna, the Pt satellites act as catalytically active reactors, and the carbon shell serves as a nanoscale dielectric spacer separating the reactors from the antenna. The precise modulation of the catalytic reaction kinetics has been systematically studied through optical excitations of the plasmon resonances in a controllable manner.

Organic liquid hydrogen carriers (OLHC) with considerable hydrogen capacity, provide an effective guide for hydrogen (H_2_) storage and transportation. Catalytic dehydrogenation of OLHC using multicomponent plasmonic hybrid nanoparticles to release H_2_ under light illumination in a mild environment, offers a feasible and promising alternative to mitigate the global environmental problems and energy crises caused by the exhaustion of fossil fuels. Li *et al.* (https://doi.org/10.1039/D3NA00663H) summarize the latest advances in plasmon-enhanced formic acid dehydrogenation at room temperature with palladium (Pd)-based hybrid plasmonic nanostructures, including geometry-dependent activities and further enhancement by coupling LSPRs with Fermi level engineering.

In recent years, the remarkable and unique attributes of chiral inorganic materials have propelled the burgeoning interest from the nanoscience community, especially chiral plasmonic nanomaterials with tunable chiroptical activities. Zhang and Sun *et al.* (https://doi.org/10.1039/D3NA00808H) discuss the recent advances in the preparation and application of multicomponent chiral plasmonic hybrid nanomaterials with emphasis on synthetic approaches as well as their emerging utilization in chirality sensing, enantioselective catalysis, and biomedicine.

Among the plasmonic nanomaterials, bimetallic nanoparticles are unique and superior in tuning physical and chemical properties compared to their monometallic counterparts because they can deliver additional functionality and/or improved stability. Ye and Ringe *et al.* (https://doi.org/10.1039/D3NA00523B) describe the synthesis of bimetallic copper (Cu) palladium (Pd) nanorods with plasmonic properties and study the Pd content effects corroborated by the numerical simulations using the electron-driven discrete dipole approximation, which paves the way for further multicomponent/multimetallic Pd nanostructures with plasmonic behavior.

The hybridization of stimuli-responsive hydrogels and plasmonic nanoparticles to form hybrid materials is another hot topic nowadays, especially in the field of multifunctional stimuli-responsive plasmonic hybrid sensors. Hanson *et al.* (https://doi.org/10.1039/D3NA00758H) report the fabrication of a thermoresponsive microgel (Au NR@PNIPMAm) by integrating Au nanorods with poly(*N*-isopropylacrylamide) and correlate the structural changes of the gel to the optical responses of the embedded plasmonic nanorods with dynamic light scattering and nuclear magnetic resonance.

As the guest editor of this themed collection, I am deeply indebted to all the dedicated authors for their timely contributions and all the reviewers for their devoted professional evaluations to help maintain the high quality of the themed issue. In addition, I would like to extend my heartfelt appreciation to the editorial staff of *Nanoscale Advances* for their generous support, especially to Dr Hannah Kerr, Deputy Editor, for her expert suggestions throughout the editing process. I sincerely hope this themed collection will serve as a platform for researchers across different disciplines to communicate and share their ideas and more importantly, provide insights into recent advances in the field of multicomponent plasmonic hybrid nanostructures, bridging the gap between fundamental research and practical applications.

## Supplementary Material
